# Do current screening recommendations allow for early detection of lithium-induced hyperparathyroidism in patients with bipolar disorder?

**DOI:** 10.1186/2194-7511-1-7

**Published:** 2013-06-14

**Authors:** Michael Berger, Michael Riedel, Nora Tomova, Michael Obermeier, Florian Seemüller, Sandra Dittmann, Hans-Jürgen Moeller, Emanuel Severus

**Affiliations:** Klinik für Psychiatrie und Psychotherapie, Ludwig-Maximilians Universität München, Nussbaumstrasse 7, Munich, 80336 Germany; Vinzenz von Paul Hospital, Abt. Psychiatrie, Schwenninger Str. 55, Rottweil, 78628 Germany; Isar-Amper Klinikum München, Ost Vockestrasse 72, Haar, 85540 Germany; Klinik und Poliklinik für Psychiatrie und Psychotherapie, Universitätsklinikum Carl Gustav Carus Technische Universität Dresden, Fetscherstr. 74, Dresden, 01307 Germany

**Keywords:** Bipolar disorder, Lithium, Hyperparathyroidism, Calcium

## Abstract

**Background:**

Current screening recommendations for early detection of lithium-associated hyperparathyroidism propose an exclusive measurement of serum albumin-adjusted calcium (Aac) concentration as a single first step. However, longitudinal data in patients with recurrent affective disorders suggest that increases in serum intact parathyroid hormone (iPTH) levels in lithium-treated patients may not necessarily be accompanied by a parallel increase in the concentration of Aac. If true, patients with an isolated increase in iPTH concentration above the reference range might be missed following current screening recommendations. Therefore, this study set out to examine key parameters of calcium metabolism, including iPTH and 25-hydroxycholecalciferol concentrations in patients with bipolar disorder that was or was not managed with lithium.

**Methods:**

Sixty patients with bipolar disorder according to DSM-IV were enrolled, 30 of whom had received long-term lithium treatment (lithium group), whereas the other 30 patients were on psychopharmacological treatment not including lithium (non-lithium group) at the time of the study. Owing to exclusion criteria (e.g., lithium < 6 months, laboratory results indicative of secondary hyperparathyroidism), 23 bipolar patients composed the final lithium group, whereas 28 patients remained in the non-lithium group for statistical analyses.

**Results:**

Patients in the lithium group showed a significantly higher concentration of iPTH compared to the non-lithium group (*p* < 0.05). Similarly, Aac concentrations were significantly increased in the lithium group compared to the non-lithium group (*p* < 0.05). However, in a multivariate linear regression model, group affiliation only predicted iPTH concentration (*p* < 0.05). In line with this, none of the four patients in the lithium group with an iPTH concentration above the reference range had an Aac concentration above the reference range.

**Discussion:**

This study suggests that the biochemical characteristics between primary hyperparathyroidism and lithium-induced hyperparathyroidism differ substantially with regard to regulation of calcium homeostasis. As such, current screening practice does not reliably detect iPTH concentrations above the reference range. Therefore, further research is needed to elucidate the consequences of an isolated iPTH concentration above the reference range in order to develop the most appropriate screening tools for hyperparathyroidism in lithium-treated patients with bipolar disorder.

## Background

Lithium is one of the most widely used and most efficacious drugs in the different treatment phases of bipolar disorders (Severus et al. 2012). In particular, recent data clearly support lithium as the first-line treatment in long-term treatment of this disorder (Weisler et al. 2011; Geddes et al. 2010; Kessing et al. 2011). However, lithium is also known to be associated with a variety of side effects including weight gain, hypothyroidism, and nephrogenic diabetes insipidus (Livingstone and Rampes 2006). Furthermore, lithium is listed as one of the causes of primary hyperparathyroidism, a biochemical syndrome characterized by increased secretion of parathyroid hormone (PTH) from one or more of the parathyroid glands (Sitges-Serra and Bergenfelz 2007; Broome and Solorzano 2011). Most cases are sporadic in nature and caused by a single adenoma (85%–95%) or multiglandular disease (5%–10%) (Sitges-Serra and Bergenfelz 2007). In case of lithium treatment, a decrease in parathyroid sensitivity to calcium has been suggested as the principal mechanism of action for the increased secretion of parathyroid hormone resulting in hypercalcemia, the biochemical hallmark of primary hyperparathyroidism (Haden et al. 1997). Consequently, monitoring calcium levels before initiating lithium therapy and every 6 months thereafter has been suggested as the primary screening parameter for lithium-induced hyperparathyroidism (Livingstone and Rampes 2006; Broome and Solorzano 2011). If calcium levels are found to be elevated, measuring intact parathyroid hormone (iPTH) levels has been recommended as the next step (Livingstone and Rampes 2006). According to the ‘International Group for The Study of Lithium Treated Patients’ , between 10% and 42% of patients on lithium therapy develop hypercalcemia and up to 29% develop hyperparathyroidism (Schleicher and Kampf 2013). The available data show that most of the studies conducted so far are case control studies, and none of the studies focus exclusively on patients with bipolar disorders in the test or control groups (Saunders et al. 2009). In general, the number of participants included in these studies is relatively small, and only a few of these studies employed parathyroid test systems complying with today's quality standards (Souberbielle et al. 2006). In addition, one of the more recent studies suggests that following 6 months of lithium treatment, patients have significantly increased parathyroid hormone concentrations without an increase in serum calcium levels (Mak et al. 1998). This ‘normocalcemic’ subtype of lithium-induced hyperparathyroidism will consequently remain unrecognized if calcium is used as the sole and primary screening instrument as is currently recommended. Interestingly, in a community-based cohort of elderly men, higher (and high normal) plasma PTH levels were associated with a higher risk of cardiovascular mortality in the absence of hypercalcemia (Hagstrom et al. 2009). Missing these cases of isolated higher parathyroid levels is of particular concern as bipolar disorder *per se* is associated with an increased risk of cardiovascular mortality (Fiedorowicz et al. 2008). Furthermore, additional studies suggest an association between elevated parathyroid hormone levels and cognitive deficits in the absence of hypercalcemia (Jorde et al. 2006; Roman et al. 2005). Therefore, we set out to answer the following hypotheses:

- Serum iPTH concentrations in patients with bipolar disorders are increased when treated with lithium compared to those patients treated with other drugs.
- Elevated iPTH concentrations above the reference range may occur without a concomitant increase in (albumin-adjusted) calcium levels.

## Methods

### Participants

Patients were recruited from the inpatient and outpatient units of the Department of Psychiatry and Psychotherapy of the Ludwig-Maximilians University, Munich. Study inclusion criteria were for patients who were (1) diagnosed with bipolar disorder (I, II) according to Diagnostic and Statistical Manual of Mental Disorders Fourth Edition (DSM-IV), (2) able to give written informed consent, and (3) between 18 and 65 years of age. In addition, for inclusion in the lithium group, patients had to have undergone lithium treatment for at least 6 months. Exclusion criteria applied to patients who had a diagnosis of current substance abuse (with or without substance dependence), borderline personality disorder or antisocial personality disorder, significant medical co-morbidity (such as diabetes, malignancies, liver disease, renal impairment, chronic infectious diseases, inflammatory bowel disease), as well as blood parameters indicative of a secondary hyperparathyroidism (reduced 25-hydroxycholecalciferol concentrations in combination with iPTH concentrations above the reference range).

### Procedures

The study was approved by the Institutional Review Board (IRB) of the Ludwig-Maximilians University, Munich. Potentially eligible patients were approached by one of the authors (MB) and informed about the study. If patients were interested in participating in this study, inclusion criteria and exclusion criteria were checked; if patients were eligible, they were asked to sign a written informed consent form. Subsequently, diagnosis was confirmed using the structured clinical interview for DSM-IV (Wittchen et al. 1997). In addition, socio-demographic data, course of the illness, and medication history were assessed by means of the Network Enrollment Questionnaire as previously used by the Stanley Foundation Bipolar Network (Suppes et al. 2001). In addition, the psychopathological state was documented using standardized rating scales (YMRS (Young et al. 1978), HAMD (Hamilton 1967), BDI (Beck et al. 1961), and Clinical Global Impressions Scale for Bipolar Illness (Spearing et al. 1997)). Finally, a blood sample was drawn 12 h after the last medication intake. The blood sample was used to determine the concentration of the following parameters: serum lithium, iPTH, calcium, albumin-adjusted calcium, phosphorus, 25-hydroxycholecalciferol, albumin, creatinine, and urea.

Parathyroid hormone concentration was measure using an electrochemiluminescence immunoassay, i.e., the Elecsys® 2010 Roche immunoassay (Roche Diagnostics, Germany) which is a reliable, standardized, and well-validated method of measuring iPTH (Hermsen et al. 2002; 2006).

### Sample size justification

Adapted from the study by Mak et al. (1998) using a variance analysis, we calculated a *p* value of <0.005 with regard to the differences in the concentration of iPTH after lithium administration. A *t* value of at least 3.62 was calculated which, in turn, allowed the determination of the effect strength ‘Cohen's *d*’ settled by 0.80. For the assessment of the difference between the two groups, a one-sided *t* test of the null hypothesis H_0_ = μLit ≤ μcontr seemed to be suitable for our investigation. As additional parameters for the power analysis, *α* = 0.05 and *β* = 0.8 were set. Given these calculations, a minimum number of 20 patients per group was estimated. Being more cautious in terms of the null hypothesis and proposing a two-sided *t* test (H_0_ = μLit = μcontr), a group strength of 26 patients was calculated. As some patients were expected to drop out after enrollment due to exclusion criteria in the blood results, a total of 30 patients per group were enrolled to ensure a sufficient number of patients in the final model.

### Statistical procedures

The data analysis was carried out using the statistical program R 2.9.0 (Hornik 2012). Clinical and demographic characteristics were compared using *t* test and Fishers exact test; *t* tests were used for metric variables. For categorical variables such as gender, bipolar (I/II), and rapid cycling (yes/no), Fishers exact test was performed.

We wanted to know if (and if so, to what extent) the parathyroid hormone concentration is affected by lithium treatment including associations with lithium levels, lithium dosage, and the duration of a lithium therapy. We also wanted to investigate whether (and if so, to what extent) calcium metabolism is altered by treatment with lithium or not. Therefore, a *t* test was used to compare both groups in terms of interesting laboratory parameters such as albumin-adjusted calcium, 25-hydroxycholecalciferol, and intact parathyroid hormone. Pearson correlation coefficients and accordant test statistics with the parathyroid hormone concentration were calculated for albumin-adjusted calcium, age, 25-hydroxycholecalciferol, lithium level, lithium dose, and duration of lithium therapy.

Selecting variables using Akaike's information criterion, two linear regression models were created that best explain the parathyroid hormone concentration and the concentration of albumin-adjusted calcium. In order to explain the response variable ‘parathyroid hormone concentration’ group affiliation, albumin-adjusted calcium, 25-hydroxycholecalciferol, age, and gender were integrated as independent variables. For the response variable ‘albumin-adjusted calcium concentration’ , parathyroid hormone, group affiliation, vitamin D, age, and gender were considered as potential explanatory variables. The goodness-of-fit was based on the adjusted *R*^2^. The average of metric variables is described as mean ± SD. For all statistical calculations, the significance level was set at 5% (*p* < 0.05).

## Results

### Study population

Thirty (30) patients with bipolar I/II disorder, who had been on lithium for at least 6 months as well as 30 bipolar patients without current lithium treatment were enrolled in the study. One patient in the lithium group and one patient in the non-lithium group were excluded due to suspected chronic kidney disease (serum creatinine concentration above the reference range). In addition, two patients in the lithium group and one patient in the non-lithium group were excluded due to suspected secondary hyperparathyroidism (parathyroid hormone concentrations above the reference range in conjunction with 25-hydroxycholecalciferol levels below the reference range). Finally, four patients in the lithium group had to be excluded as new information emerged, indicating that they had been on lithium for less than 6 full months. Therefore, a total of 23 patients on psychopharmacological treatment (including lithium) and 28 patients on psychopharmacological treatment (excluding lithium) were included in the statistical analysis.

The demographic and clinical characteristics of both groups are shown in Table 1. The only item which differed significantly between the groups was the total score of the 21-item HAMD scale. According to this scale, patients in the non-lithium group (7.86 ± 5.86) were more severely depressed than those in the lithium group (4.61 ± 3.13) (*p* = 0.02).

**Table 1:** **Demographic and clinical variables of the study population**

	Total	Lithium	Non-lithium	***p*** value
	(***n*** = 51)	(***n*** = 23)	(***n*** = 28)	
Age (years), mean ± SD	46.57 ± 9.21	48.48 ± 8.98	45 ± 9.26	0.18
Gender (male/female)	31/20	14/9	17/11	1
Bipolar (I/II)	33/18	12/11	21/7	0.14
Age at onset (years), mean ± SD	25.14 ± 11.24	24.43 ± 10.86	25.71 ± 11.71	0.69
Number depressive episodes (mean ± SD)	12.98 ± 15,79	10.55 ± 11,69	15.04 ± 18.56	0.31
Number mania/hypomania (mean ± SD)	8.87 ± 11.78	6.62 ± 9.11	10.76 ± 13.53	0.22
Number of drugs (mean ± SD)	2.41 ± 1.22	2.57 ± 1.20	2.29 ± 1.24	0.42
Rapid cycling (no/yes)	46/5	22/1	24/4	0.35
HAMD (mean ± SD)	6.39 ± 5.05	4.61 ± 3.13	7.86 ± 5.86	0.02
BDI (mean ± SD)	32 ± 8.84^a^	29.39 ± 5.66	33.50 ± 10.93^a^	0.09
YMRS (mean ± SD)	2.10 ± 2,56	2.04 ± 2,01	2.14 ± 2.98	0.88

^a^One missing value. HAMD, Hamilton depression scale; BDI, Beck Depression Inventory; YMRS, Young Mania Rating Scale.

The mean number of drugs was 2.57 ± 1.20 in the lithium group, compared to 2.29 ± 1.24 in the non-lithium group (*p* = 0.42). Among the patients in the lithium-group, 13 were treated with anticonvulsants (valproic acid, *n* = 2; lamotrigine, *n* = 9; carbamazepine, *n* = 1; levetiracetam, *n* = 1), while 24 patients in the non-lithium group were treated with anticonvulsants (valproic acid, *n* = 8; lamotrigine, *n* = 16).

### Intact parathyroid hormone levels and albumin-adjusted calcium concentrations

The mean intact parathyroid hormone concentration of all 51 bipolar patients was 42.02 ± 16.81 pg/ml. The mean concentration in the lithium group was 47.67 ± 18.27 pg/ml, compared to 37.38 ± 14.20 pg/ml in the non-lithium group (*p* = 0. 03) (Table 2).

**Table 2:** **Laboratory parameters of the study population**

	Lithium	Non-lithium	***p*** value
	(***n*** = 23)	(***n*** = 28)	
iPTH concentration (mean ± SD)	47.67 pg/ml ± 18.27	37.38 pg/ml ± 14.20	0.03
	range 26.50 to 92	range 14.80 to 66.50	
Albumin-adjusted calcium concentration (mean ± SD)	2.35 mmol/l ± 0.10	2.29 mmol/l ± 0.10	0.045
	range 2.18 to 2.62	range 2.14 to 2.57	
25-hydroxycholecalciferol concentration (mean ± SD)	23.65 ng/ml ± 9.20	24.56 ng/ml ± 7.85	0.71
	range 6.40 to 45.80	range 13.20 to 43.70	

Four (4) out of 23 patients (17.39%) in the lithium group showed parathyroid hormone concentrations above the reference range (15 to 65 pg/ml) compared to two of the 28 patients in the non-lithium group (7.14%). In all six patients with iPTH levels above the reference range, Aac concentrations were found within the normal range.

The mean concentration of Aac in the lithium group was 2.35 ± 0.10 mmol/l, compared to 2.29 ± 0.10 mmol/l in the non-lithium group (*p* = 0.045). In the lithium group, one patient showed albumin-adjusted calcium above the reference range without an accompanying increase in parathyroid hormone. No patient in the non-lithium group showed elevated concentrations for albumin-adjusted calcium.

**Figure 1:**
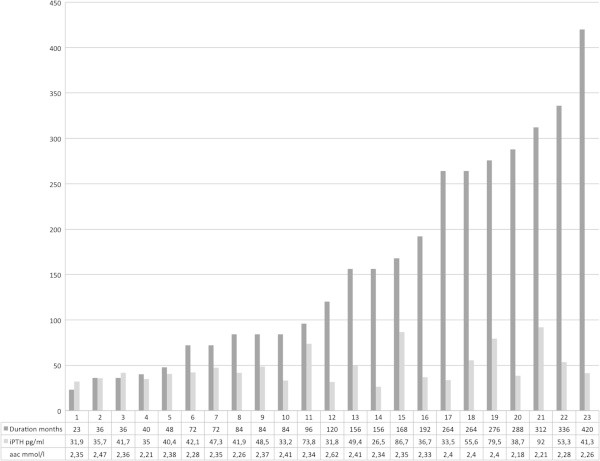
**Duration of lithium treatment (in ascending order), iPTH concentration, and albumin-adjusted calcium levels.** The data represent every single patient in the lithium group.

In the lithium group, no significant correlation between the levels of iPTH and Aac concentration could be detected (correlation coefficient = −0.16, *p* = 0.48). Similarly, there were no significant correlations between iPTH and 25-hydroxycholecalciferol (correlation coefficient = 0.04, *p* = 0.84) or age (correlation coefficient = 0.12, *p* = 0.59). In the non-lithium group there was no significant correlation between iPTH level and Aac concentration either (correlation coefficient = 0.27, *p* = 0.16). In addition, there was no significant correlation between iPTH concentrations and lithium level (correlation coefficient = 0.03, *p* = 0.89), lithium dose (correlation coefficient = 0.36, *p* = 0.09), or duration of lithium treatment (correlation coefficient = 0.34, *p* = 0.11) in the lithium group (Figure 1).

Finally, a multivariate linear regression model was performed with ‘iPTH concentration’ being the dependent variable, and group affiliation (lithium versus non-lithium, respectively), age, gender, 25-hydroxycholecalciferol concentration, and Aac concentration being the explanatory variables. Using a forward-backward stepwise selection, ‘group affiliation’ remained the only explanatory variable showing statistical significance (regression coefficient = 0.25, *p* = 0.03). The goodness-of-fit (adjusted *R*^2^) was 0.08.

Similarly, a multivariate linear regression model was performed with ‘Aac concentration’ being the dependent variable, and iPTH concentration, group affiliation, 25-hydroxycholecalciferol concentration, age, and gender being the explanatory variables. Using forward-backward stepwise selection only, ‘group affiliation’ emerged as a significant factor (regression coefficient = 0.0646, *p* = 0.0231). The goodness-of-fit (adjusted *R*^2^) was 0.08.

## Discussion

To the best of our knowledge, this is the first study to systematically assess the role of lithium maintenance treatment on calcium metabolism, including iPTH levels and 25-hydroxycholecalciferol concentrations in patients with bipolar disorder. In our study, iPTH levels were significantly higher in patients with bipolar disorder undergoing psychopharmacological treatment that included lithium for at least 6 months (lithium group) compared to patients with bipolar disorder receiving psychopharmacological treatment that excluded lithium (non-lithium group). In line with this, group affiliation (lithium versus non-lithium) was the only variable explaining iPTH concentrations. Furthermore, none of our patients with iPTH concentrations above the reference range would have been detected using current screening recommendations (Livingstone and Rampes 2006; Saunders et al. 2009).

Our findings support the systematic review by Saunders et al. (2009), which indicates that lithium may cause an increase in serum calcium levels. Our study also supports the results by Mak et al. (1998), which demonstrated that patients with affective disorders on lithium therapy show significantly elevated parathyroid hormone levels starting at 6 months of lithium treatment and maintaining them until the end of the study at 24 months compared to baseline. In contrast to Mak et al. (1998), in our study, Aac concentrations were also significantly increased in the lithium group compared to the non-lithium group. However, in the study by Mak et al. (1998), Aac concentration was numerically highest at the end of the study period (i.e., at 24 months). In our study, the patients in the lithium group were on lithium for an average of 157.70 ± 114.64 months. Therefore, with increasing length of lithium intake, the hypercalcemic properties of iPTH may dominate and may have contributed to this significant increase in Aac concentrations compared to the control group (non-lithium treated patients) in our study. In addition, neither Aac concentrations were increased in individuals with elevated iPTH levels nor was there a positive significant correlation between iPTH levels and Aac concentrations in the lithium group, as has been shown in lithium-independent normocalcemic or hypercalcemic primary hyperparathyroidism (Shlapack and Rizvi 2012; Silverberg and Bilezikian 2003). In contrast, calcium levels were exclusively predicted by group affiliation. These findings strongly argue in favor of the idea, as suggested by Mak et al. (1998), that lithium may modulate the direct hypercalcemic action of iPTH by its iPTH-independent direct actions on organs affecting calcium metabolism, such as the kidney and the bones. In the study by Mak et al. (1998), fasting urinary calcium excretion was decreased throughout the study period compared to the baseline. As alkaline phosphatase was unchanged, this may hint to decreased bone resorption (the opposite of the expected physiological effect of increased iPTH levels) and may have contributed to the statistically unchanged Aac concentrations throughout the study period. In this context, it seems noteworthy that in a case–control study, adjusted for psychotropic drug use, lithium treatment was associated with a decreased risk of fractures potentially pointing at bone anabolic properties (Vestergaard et al. 2005).

In line with lithium's iPTH-independent actions on calcium metabolism, our study suggests that measuring Aac concentrations is an unsuitable tool to reliably detect increased parathyroid hormone levels in lithium-treated patients with bipolar disorder. This is in clear contrast to current screening recommendations regarding hyperparathyroidism in lithium-treated patients (Livingstone and Rampes 2006; Saunders et al. 2009). Whether the current screening practice needs to be changed in which iPTH levels be used as primary screening tool will depend on whether isolated iPTH level increases have negative consequences on human health, as some data, in particular with regard to mood (Hoogendijk et al. 2008), cognition (Jorde et al. 2006; Roman et al. 2005), and cardiovascular disease (Hagstrom et al. 2009), suggest.

This study has several limitations:

The present study is a cross-sectional case–control study examining parathyroid hormone concentrations in patients with bipolar disorder with or without a current mood episode, treated with an average of 2.5 different drugs, with lithium being part of this treatment regimen in one of the two groups. Ideally, to answer the question whether lithium causes an increase in parathyroid hormone levels in patients with bipolar disorder, a randomized, double-blind, placebo-controlled trial would be needed in which euthymic patients with bipolar disorder are either randomized to receive lithium monotherapy or placebo. Unfortunately, in addition to economic reasons, such a trial is unlikely to happen in the near future as keeping patients with bipolar disorder stable on placebo for at least 6 months represents a true clinical challenge; the probability of relapse/recurrence within this period of time is substantial and depriving patients from psychopharmacological treatment indicated according to current treatment guidelines is difficult to justify from an ethical point of view. Alternatively, randomizing bipolar patients to either lithium or another approved long-term treatment may be more feasible from a clinical point of view. However, approved long-term treatment options for bipolar disorder other than lithium are either known to interact with calcium metabolism and parathyroid hormone secretion or unknown (Kim et al. 2007; Ali et al. 2004).

In our study, no significant correlation was found between iPTH concentration and lithium level, lithium dose, or duration of lithium treatment. This may be due to a variety of reasons. First, this study was not powered to answer these issues, e.g., the number of patients in the lithium group may have been too small. Second, while clinical practice suggests that elevated levels of calcium (and iPTH) primarily seem to appear after many years of lithium treatment, very little is known about iPTH levels before calcium levels have been found to be elevated on the occasion of periodical assessment because they simply were not assessed. Third, when elevated levels of calcium and iPTH have emerged after many years of lithium treatment, the treating psychiatrists may switch the patient to another drug for prophylactic treatment not associated with this type of side effect. Therefore, only those individuals who do not develop this type of side effect after many years of treatment should continue on lithium and had the chance to be part of the lithium group in our study.

The majority of patients in our lithium group took 2.57 different psychopharmacological drugs at the point of assessment. While this number is not unusual in today's published research samples of bipolar disorders (Goldberg et al. 2009), this study does not address - and therefore cannot answer - the question whether or not the results would be the same in patients with bipolar disorders fully responding to long-term lithium monotherapy, as they tend to experience fewer side effects on lithium than those not fully responding (Grof et al. 1993).

Given the cross-sectional design used as well as the present sample size, we were unable to answer other important questions related to the subject of our paper, such as the natural course of calcium levels and iPTH concentrations during long-term lithium treatment, the impact geography might play, and the effects of dosage reduction on the aforementioned parameters. These issues should be addressed in future studies.

In this study, we excluded patients with laboratory results indicative of secondary hyperparathyroidism (parathyroid hormone concentrations above the reference range in conjunction with 25-hydroxycholecalciferol levels below the reference range) from our analyses. However, this is unlikely to have a major impact on the present results as just two patients in the lithium group and one patient in the non-lithium group had to be excluded for this reason. Furthermore, in our final sample, 25-hydroxycholecalciferol concentrations neither differed significantly between the groups nor did they explain parathyroid hormone concentrations.

HAMD depression scores were significantly increased in the non-lithium group compared to the lithium group. This is most likely due to the fact that patients on lithium treatment for at least 6 months were predominantly found among euthymic outpatients. While affective symptoms are not known to have an impact on iPTH levels; increased iPTH levels have been associated with depression in adults in some (Hoogendijk et al. 2008; Driessen et al. 1995), but not all studies (Zhao et al. 2010; Jaddou et al. 2012). Therefore, higher depression scores are unlikely to have contributed to the increase in iPTH levels in the lithium group.

Similarly, anticonvulsants, including lamotrigine and valproic acid, have been shown to increase parathyroid hormone levels in patients with epilepsy (Kim et al. 2007). Therefore, the differences in parathyroid hormone levels found in our study are unlikely to be brought about by the higher number of patients in the non-lithium group being on valproic acid or lamotrigine.

In our study, there was a substantial discrepancy between the severity of the depressive syndrome judging from the HAMD and BDI, respectively. However, this has been repeatedly reported in the literature in the same direction as in our study and may, at least partly, be explained by the HAMD being more sensitive to change in psychopathology (Schneibel et al. 2012) and personality characteristics, such as high neuroticism and low extraversion, which may further add to this discrepancy (Enns et al. 2000).

## Conclusions

This study strongly suggests that patients with bipolar disorder who have undergone lithium treatment for 6 months or more show an increase in iPTH and Aac concentrations compared to psychopharmacological treatment without lithium. In addition, in contrast to primary hyperparathyroidism, screening with Aac concentrations does not allow reliable detection of individual patients with iPTH concentrations above the reference range. Whether the current screening practice needs to be changed and iPTH levels be used as a primary screening tool will depend on whether isolated iPTH level increases have negative consequences on human health, as some preliminary data (in particular with regard to mood, cognitive function, and cardiovascular disease) suggest. To better understand the consequences of an isolated increase in iPTH concentration, the impact of parathyroid lowering drugs such as cincacalcet (Gregoor and de Jong 2007; Sloand and Shelly 2006; Hong et al. 2012) or 25-hydroxycholecalciferol (if levels are suboptimal) (Durazo-Arvizu et al. 2010) on mood and/or cognitive symptoms in lithium-treated symptomatic patients with bipolar disorder should be studied in a randomized, controlled fashion.

## Abbreviations

Aac: Albumin-adjusted calcium
BDI: Beck Depression Inventory
DSM-IV: Diagnostic and Statistical Manual of Mental Disorders Fourth Edition
HAMD: Hamilton Depression Scale
iPTH: Intact parathyroid hormone
PTH: Parathyroid hormone
YMRS: Young Mania Rating Scale.
